# Review and integrative taxonomy of the genus *Prosopistoma* Latreille, 1833 (Ephemeroptera, Prosopistomatidae) in Thailand, with description of a new species

**DOI:** 10.3897/zookeys.825.32443

**Published:** 2019-02-27

**Authors:** Boonsatien Boonsoong, Michel Sartori

**Affiliations:** 1 Animal Systematics and Ecology Speciality Research Unit (ASESRU), Department of Zoology, Faculty of Science, Kasetsart University, Bangkok 10900, Thailand Kasetsart University Bangkok Thailand; 2 Museum of Zoology, Palais de Rumine, Place Riponne 6, CH-1005 Lausanne, Switzerland Museum of Zoology Lausanne Switzerland; 3 Department of Ecology and Evolution, Lausanne University, CH-1015 Lausanne, Switzerland Lausanne University Lausanne Switzerland

**Keywords:** *
Prosopistoma carinatum
*, *
Prosopistoma annamense
*, *
Prosopistoma sinense
*, COI, mayfly

## Abstract

Three species of the genus *Prosopistoma* Latreille, 1833 (Prosopistomatidae) are currently reported from Thailand. A new species, *Prosopistomacarinatum***sp. n.**, is described here based on specimens from western and southern Thailand. The new species can be easily distinguished from the other members of *Prosopistoma* by the following combination of characteristics: (i) the presence of two ridged longitudinal lines on each side of its carapace, (ii) antenna 7-segmented, (iii) a strongly convex carapace and (iv) nine pectinate setae on the ventral margin of the fore tibiae. A comparison between the key characteristics of *P.carinatum***sp. n.** and the known Thai species is provided. Results of analysis of the mitochondrial cytochrome oxidase I (COI) gene (658 bp) of three species, as well as the distribution of the Thai species, are also discussed.

## Introduction

Prosopistomatidae (Ephemeroptera) is a monogeneric family, represented by the genus *Prosopistoma*, which was originally established by Latreille (1833). Of the 27 species described to date, 16 species have been described from the Oriental region (Lieftinck 1932, Peters 1967, Soldán and Braasch 1984, Tong and Dudgeon 2000, Sartori and Gattolliat 2003, Zhou and Zheng 2004, Barber-James et al. 2008, Barber-James 2009, Shi and Tong 2013; Balachandran et al. 2016, Roopa et al. 2017). An updated key to the known species of this realm was provided by Shi and Tong (2013).

In Thailand, only the larvae of *Prosopistomaannamense* Soldán & Braasch, 1984, *P.sinense* Tong & Dudgeon, 2000, and *P.wouterae* Lieftinck, 1932 are known (Parnrong et al. 2002, Tungpairojwong and Boonsoong 2011). In the present study, we describe a new species of *Prosopistoma* based on specimens from western and southern Thailand. In addition, a distribution map of the genus in Thailand and mitochondrial COI sequence data are provided.

## Materials and methods

The prosopistomatid mayfly larvae were collected from streams and rivers in northern, north-eastern, eastern, central, western, and southern Thailand from 2013 to 2018. Measurements (given in mm) and photographs were taken using a Visionary LK System (Dun, Incorporated, USA). All drawings were made with the aid of a camera lucida attached to a compound microscope. For Scanning electron microscopy (SEM), specimens (head, carapace, sternal plate, foreleg) were dried in a Critical Point Drier (CPD7501) and coated with gold (10 nm, Sputter Coater SC7620). SEM photographs were observed by a FEI Quanta 450 SEM. Final plates were prepared with Adobe Photoshop CC 2017. The material is deposited in the collection of the Zoological Museum at Kasetsart University in Bangkok, Thailand (**ZMKU**) and at the Museum of Zoology in Lausanne, Switzerland (**MZL**). The distribution map was generated via the Simple Mapper website (http://www.simplemappr.net) using GPS coordinates.

The collected specimens were fixed in absolute ethanol and preserved under refrigeration for description and DNA extraction. Details of the specimens from the three species used for the DNA experiment are shown in Table 1. Total DNA extraction was performed using a genomic DNA purification kit (NucleoSpin, Macherey-Nagel, Germany) following the manufacturer’s protocol. A fragment of the mitochondrial gene cytochrome oxidase I (COI) gene was amplified (658 bp) using the primers LCO1490 (5'-GGT CAA ATC ATA AAG ATA TTG G-3') and HCO2198 (5'-TAA ACT TCA GGG TGA CCA AAA AAT CA-3'), designed by Folmer et al. (1994). Polymerase chain reaction (PCR) conditions were as follows: a 50 μl final reaction volume containing 25 μl of PCR Master mix solution, 1 μl (10 μm) of each primer, 2 μl of total DNA and 21 μl of nuclease free sterile water. PCR was performed as follows: 5 minutes at 94 °C, then 30 seconds at 94 °C, 30 seconds at 48 °C, and 60 seconds at 72 °C (40 cycles), and a final elongation step at 72°C for 10 minutes (Gattolliat et al. 2015). Purification and sequencing were conducted by Macrogen, Inc. (South Korea). Sequence alignment and editing were performed using ClustalW. The best-fit evolution model obtained was T92 (Tamura 3-parameter) + G. Phylogenetic trees based on maximum likelihood (ML) were performed with MEGA 7 using the likelihood-ratchet method with 1,000 bootstrap replicates. Pairwise (uncorrected-p) sequence distances were also calculated using MEGA 7 (Kumar et al. 2016). Nucleotide sequences obtained in this study have been deposited in the GenBank database (MK285321-MK285330).

**Table 1.** List of the sequenced specimens.

Species	Code	Collection locality	Collector	Date	GenBank accession number
* P. annamense *	PA-01-KN	Kanchanaburi	B. Boonsoong	15 Oct 2015	MK285321
	PA-12-RT	Ratchaburi	B. Boonsoong	13 Feb 2016	MK285322
	PA-13-LE	Loei	B. Boonsoong	19 Mar 2016	MK285323
	PA-17-NN	Nakhon Nayok	B. Boonsoong	25 Feb 2017	MK285324
	PA-18-CT	Chantaburi	B. Boonsoong	5 Jul 2018	MK285325
*P.carinatum* sp. n.	PC-01-KN	Kanchanaburi	B. Boonsoong	26 Apr 2014	MK285326
	PC-02-KN	Kanchanaburi	B. Boonsoong	26 Apr 2014	MK285327
* P. sinense *	PS-01-KN	Kanchanaburi	B. Boonsoong	20 Feb 2016	MK285328
	PS-02-KN	Kanchanaburi	B. Boonsoong	20 Feb 2016	MK285329
	PS-03-CM	Chaing Mai	B. Boonsoong	11 Mar 2017	MK285330

### Abbreviations

**C** central;

**m** meter;

**N** northern;

**NE** north-eastern;

**W** western.

## Taxonomy

### Family Prosopistomatidae Lameere, 1917

#### Genus *Prosopistoma* Latreille, 1833

##### Prosopistoma annamense

Taxon classificationAnimaliaEphemeropteraProsopistomatidae

Soldán & Braasch, 1984

**Figure 1.**
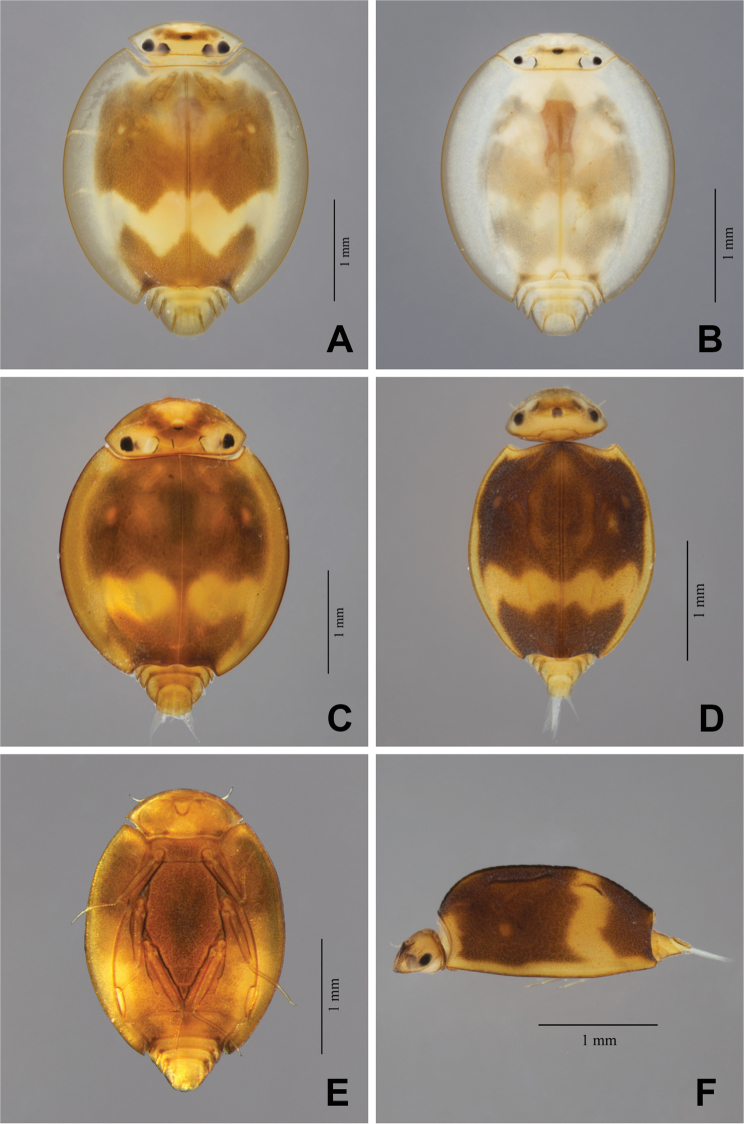
*Prosopistoma* spp. whole larvae: **A–B***P.annamense*, specimens from stream (**A**) and river (**B**); **C***P.sinense***D–F***P.carinatum* sp. n. **D** dorsal view **E** ventral view **F** lateral view.

**Figure 2.**
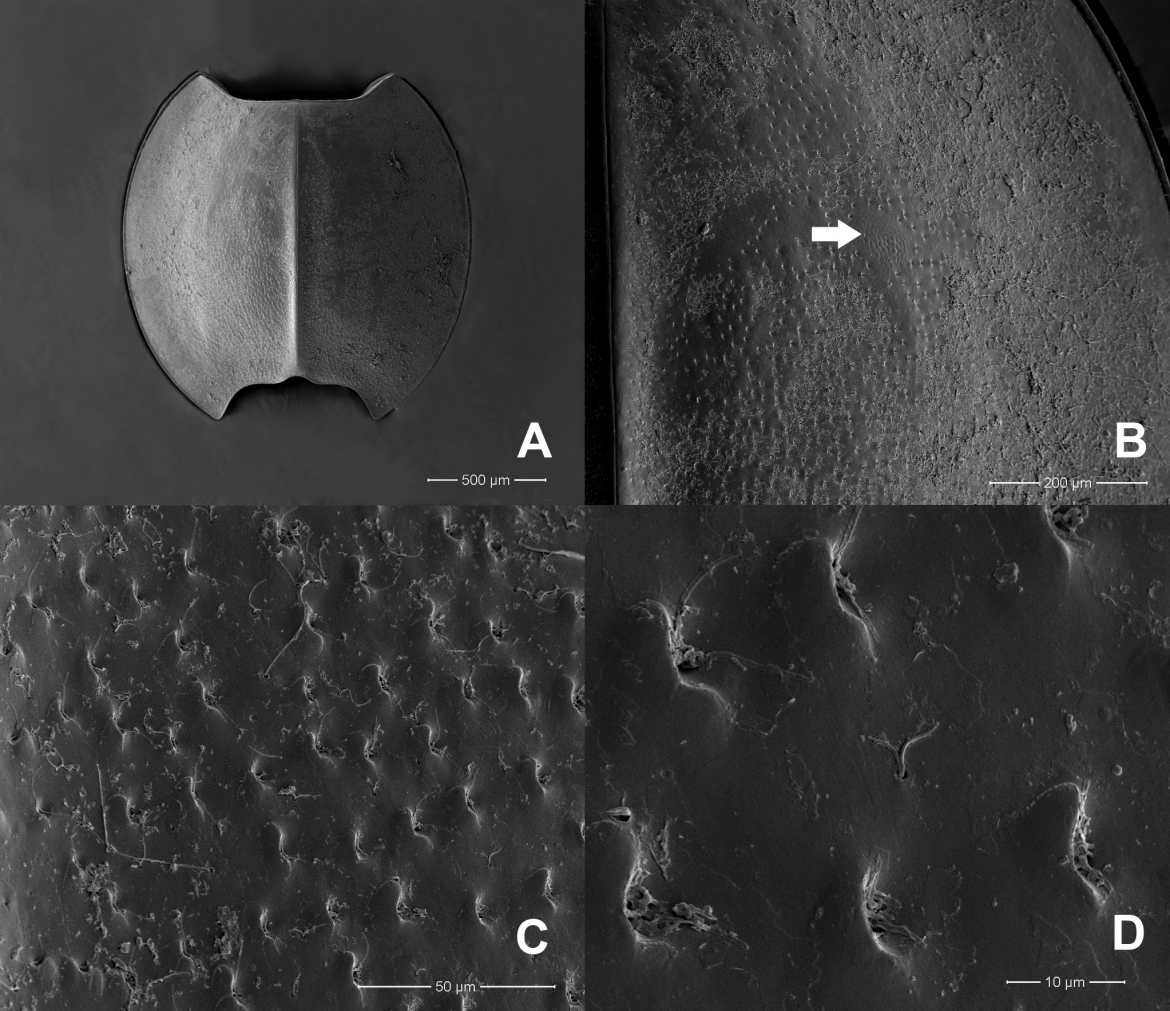
*Prosopistomaannamense*: **A**SEMs of notal shield, dorsal view **B** pale-coloured areas on carapace (arrow) **C** scale-like structures **D** closer view showing details of scale-like structures.

**Figure 3.**
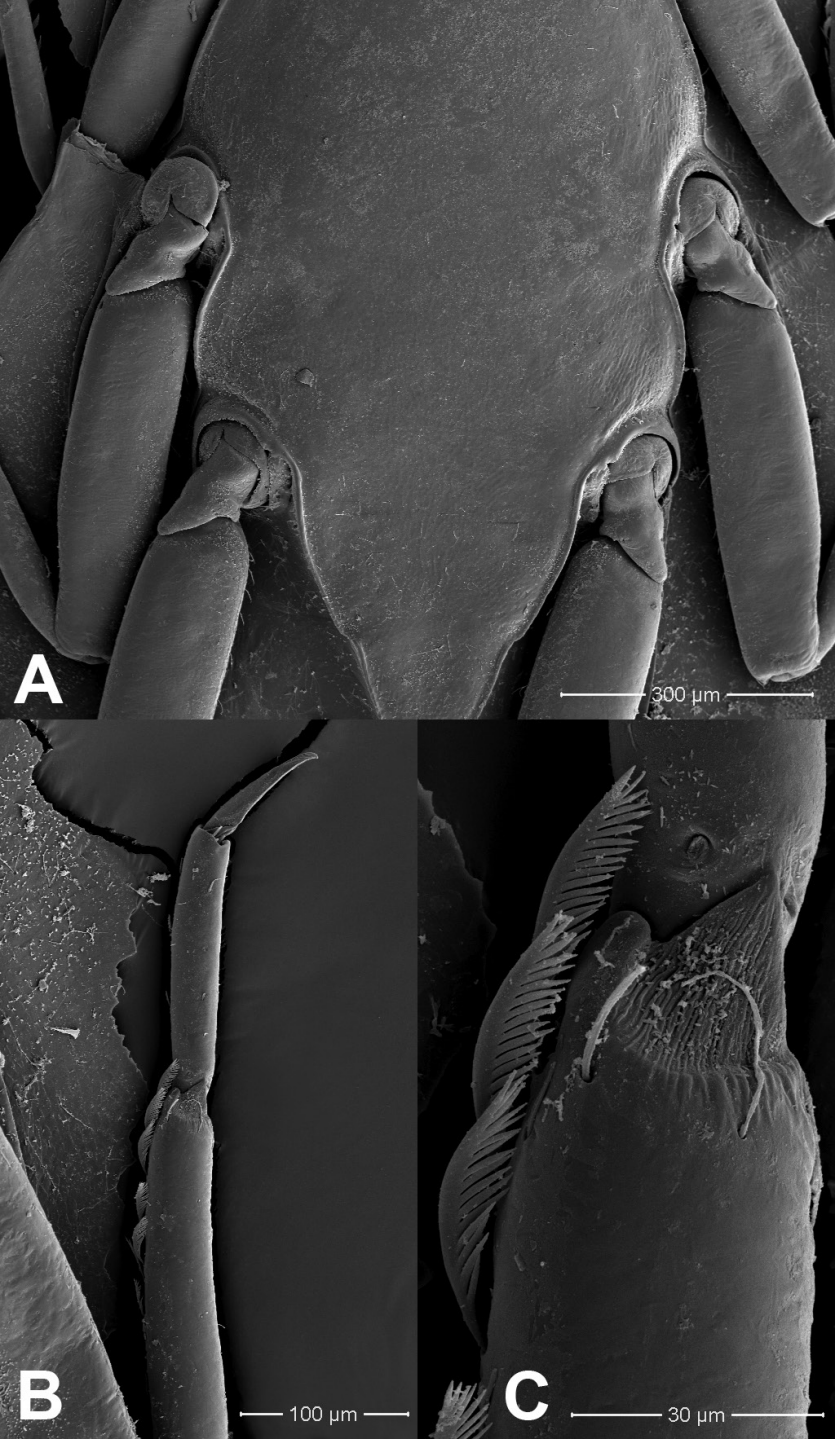
*Prosopistomaannamense*: **A**SEMs of sternal plate **B** ventral margin of fore tibia **C** pectinate setae.

Figures 1A , 1B , 2A , 2B , 2C , 2D , 3A , 3B , 3C , 12A , 12B , 12C , 14

Prosopistoma annamense Soldán & Braasch, 1984: 370–376, figs. 2, 4, 6, 8, 13, 14. (orig.); Barber-James, 2009: 153–154, Table 2 (morph. matrix); Tungpairojwong & Boonsoong, 2011: 67–68; Shi & Tong, 2013: 95 (key).

###### Material examined.

THAILAND; 1 larva, Chanthaburi province, Klong Kla Seu Yai, Khao Kitchakut, 12°52'35.94"N, 102°05'48.3"E, 40 m, 7.II.2013, 2 larvae, same place, 6.VII.2018, B Boonsoong leg. (ZMKU); 1 larva, Nakhon Nayok province, Wang Ta Krai, 14°19'35.9"N, 101°18'05.9"E, 65 m, 24.III.2013, B Boonsoong leg. (ZMKU); 5 larvae, Kanchanaburi province, Thong Pha Phum, Phu Iyara Resort, 14°37'34.4"N, 98°34'17.0"E, 207 m, 6.IV.2013, B Boonsoong leg. (ZMKU), 1 larva same data (MZL, GBIFCH00657966); 1 larva, Ban Pra Chum Mai, 14°35'01.4"N, 98°34'54.3"E, 233 m, 26.IV.2014, B Boonsoong leg. (ZMKU); 5 larvae, Kanchanaburi province, Pung Wan Resort, 14°12'20"N, 98°03'36"E, 28 m, 14.X.2015, B Boonsoong leg. (ZMKU), 1 larva same data (MZL, GBIFCH00657965); 3 larvae, Loei province, Ban Non Pattana, 17°06'24"N, 101°28'44"E, 530 m, 1.II.2016, B Boonsoong leg. (ZMKU); 3 larvae, Ratchaburi province, Kang Som Maew, Suan Phueng, 13°24'27.6"N, 99°16'51.3"E, 206 m, 13.II.2016, B Boonsoong leg. (ZMKU); 6 larvae, Loei province, Ban Non Pattana, 17°06'24"N, 101°28'44"E, 530 m, 19.III.2016, B Boonsoong leg. (ZMKU); 2 larvae, Nakhon Nayok province, Wang Ta Krai, 14°20'9.42"N, 101°18'22.5"E, 67 m, 25.II.2017, B Boonsoong leg. (ZMKU); 2 larvae, Chanthaburi province, Klong Sa Tor Bon, 12°43'12.42"N, 102°23'19.26"E, 115 m, 5.VII.2018, B Boonsoong leg. (ZMKU).

###### Diagnosis.

The larvae of *P.annamense* can be distinguished from those of other Oriental congeners by (i) apex of the inner margin of the fore tibiae, with 4–6 serrated pectinate spines (Fig. 3B), (ii) three long, finely serrated bristles at the base of the inner incisor, on both right and left mandible, (iii) segment III of the maxillary palp slightly shorter than 1/3 the length of segment II and (iv) posterolateral spines at segments VII and VII parallel to or bent outwards from the body axis (Fig. 1A, B).

###### Distribution.

Loei province (NE), Nakhon Nayok province (C), Chanthaburi province (E), Kanchanaburi province (W), Ratchaburi province (W).

###### Remarks.

The larvae of *P.annamense* were originally described by Soldán and Braasch (1984) and collected from Vietnam (Thuan Hai province). They are widely distributed in southern and central Vietnam. Tungpairojwong and Boonsoong (2011) reported this species in middle and moderately disturbed streams in Loei province (NE), Chaiyaphum province (NE) and Kanchanaburi province (W). In this study, we found this species in several provinces, and it seems to have a wide distribution in Thailand. Surprisingly, we found larvae on the banks of the Khwae Noi River (Kanchanaburi province) (Fig. 12C). This is the first report of *Prosopistoma* larvae in large rivers in Thailand, and the larvae collected from large rivers are paler than those larvae from streams (Fig. 1B).

**Table 2.** Uncorrected pairwise genetic distances (COI) between selected prosopistomatid and baetiscid species, using the Kimura 2-parameter.

	* B. laurentina *	* P. annamense *	*P.carinatum* sp. n.	* P. sinense *
* B. laurentina *	–	–	–	–
* P. annamense *	0.32	–	–	–
*P.carinatum* sp. n.	0.23	0.31	–	–
* P. sinense *	0.34	0.33	0.35	–

##### Prosopistoma sinense

Taxon classificationAnimaliaEphemeropteraProsopistomatidae

Tong & Dudgeon, 2000

**Figure 4.**
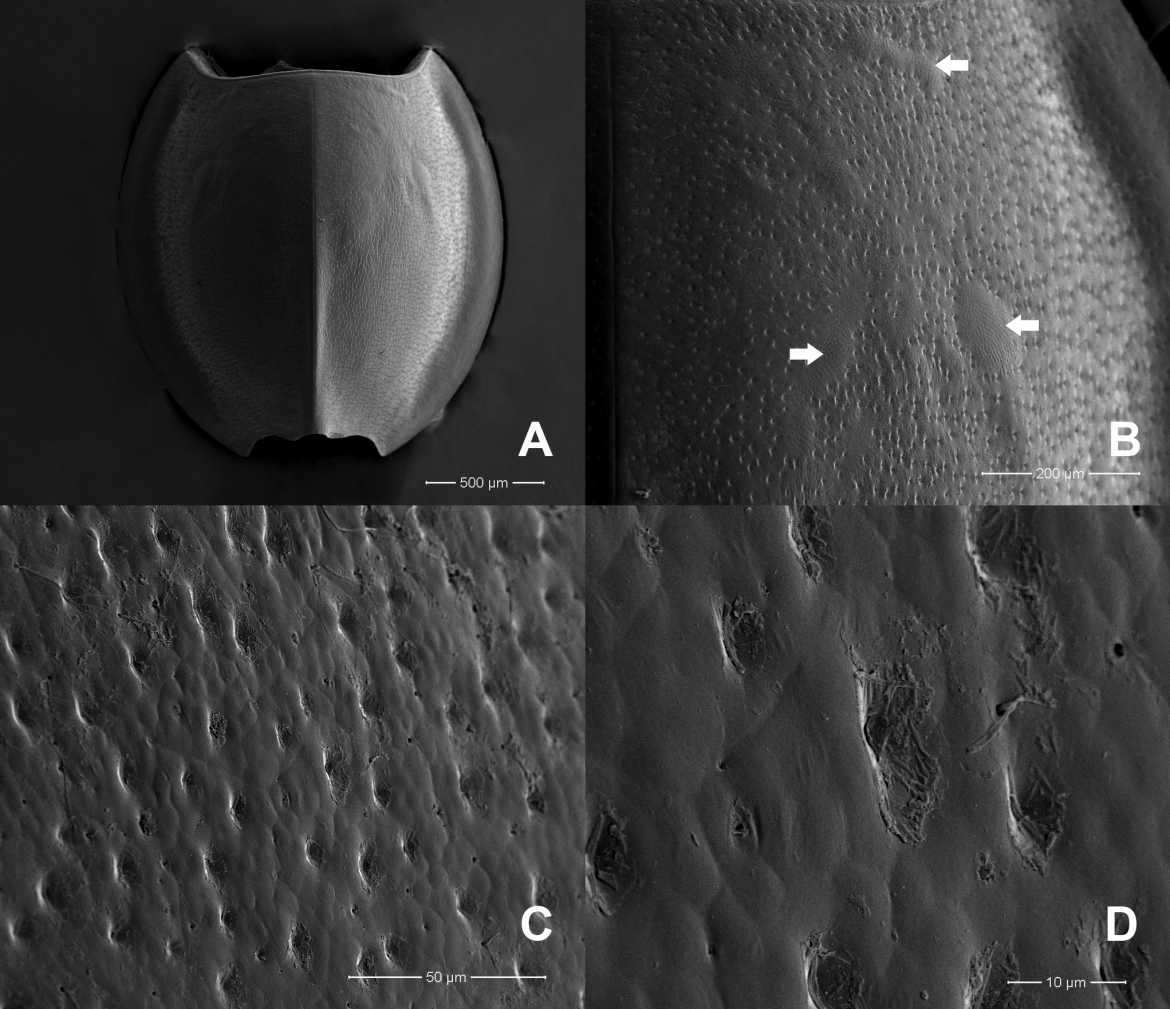
*Prosopistomasinense*: **A**SEMs of notal shield, dorsal view **B** pale-coloured areas on carapace (arrow) **C** scale-like structures **D** closer view showing details of scale-like structures.

**Figure 5.**
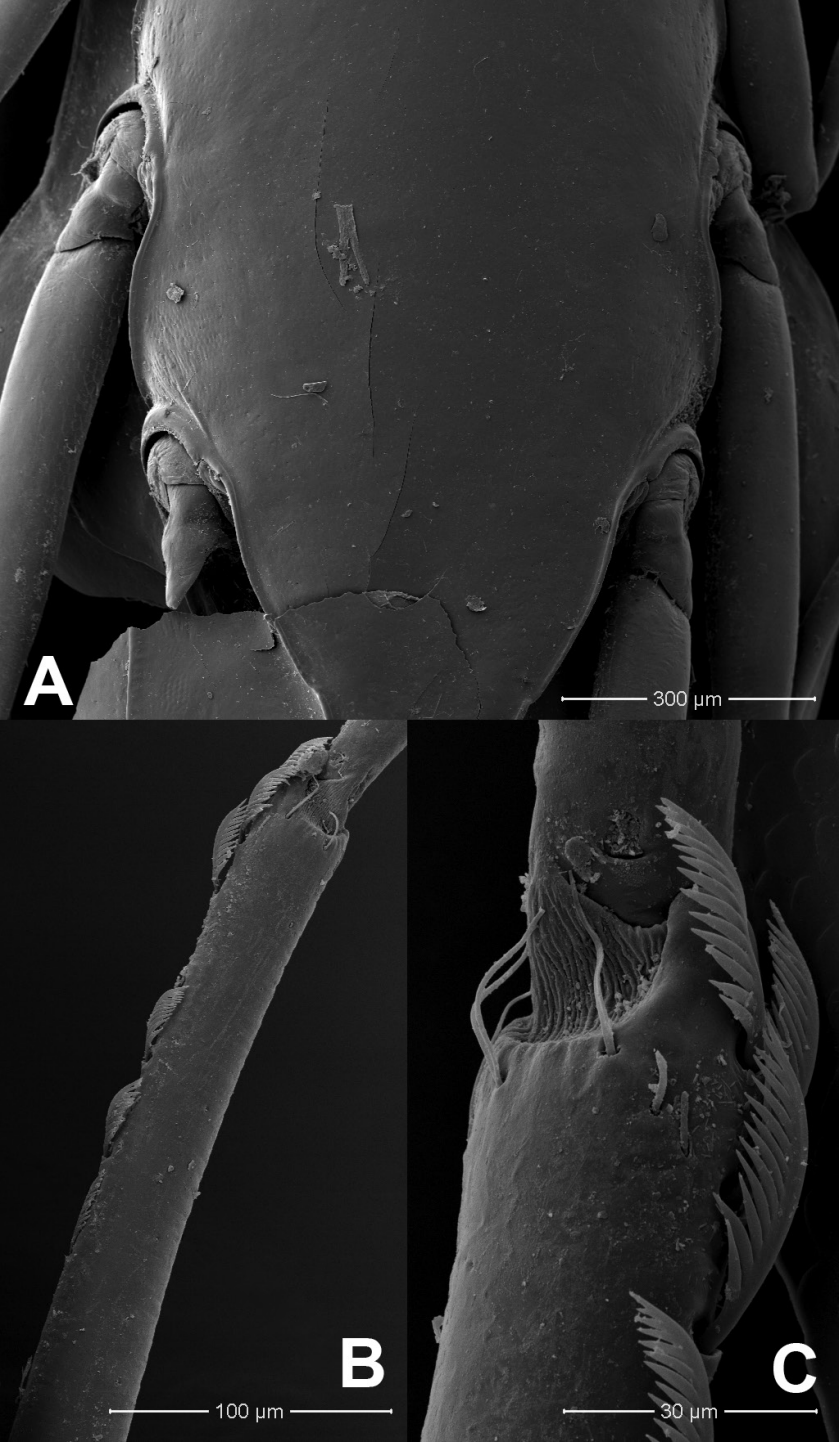
*Prosopistomasinense*: **A**SEMs of sternal plate **B** ventral margin of fore tibia **C** pectinate setae.

Figures 1C , 4A , 4B , 4C , 4D , 5A , 5B , 5C , 12D , 14

Prosopistoma sinense Tong & Dudgeon, 2000: 122–128, figs. 1–14. (orig.); Barber-James, 2009: 153–154, Table 2 (morph. matrix); Tungpairojwong & Boonsoong, 2011: 68; Shi & Tong, 2013: 95 (key).

###### Material examined.

THAILAND; 2 larvae, Kanchanaburi province, Thong Pha Phum, Pussaduklang Ranger Station, 14°33'13"N, 98°34'17"E, 317 m, 20.II.2016, B Boonsoong leg. (ZMKU); 2 larvae, Kanchanaburi province, Thong Pha Phum, Pak Kok stream, 14°39'32.1"N, 98°31'59.2"E, 161 m, 20.II.2016, B Boonsoong leg. (ZMKU), 1 larva same data (MZL, GBIFCH00657969); 4 larvae, Chiang Mai province, Chiang Dao, Mae Na, 19°19'13.08"N, 98°53'25.98"E, 742 m, 11.III.2017, B Boonsoong leg. (ZMKU).

###### Diagnosis.

The larvae of *Prosopisomasinense* can be distinguished from those of other congeners by (i) antennae with 4–5 segments, (ii) antenna segment III much longer than the combined length of segments IV–V (iii) and apex of the ventral margin of the fore-tibia with 6–8 serrated spines (Fig. 4B) (Shi and Tong 2013).

###### Distribution.

Kanchanaburi province (W), Chiang Mai province (N).

###### Remarks.

The larvae of *P.sinense* were originally described by Tong and Dudgeon (2000) and collected from China (Guangdong, Hongkong). In Thailand, Tungpairojwong and Boonsoong (2011) reported this species from a slightly disturbed stream in Kanchanaburi province. In this study, we found the larvae in the same habitat (Fig. 12D) and streams as previous studies. In addition, we found this species at a slightly disturbed stream in Chiang Dao, Chiang Mai province (N).

##### Prosopistoma wouterae

Taxon classificationAnimaliaEphemeropteraProsopistomatidae

Lieftinck, 1932

Figure 14

Prosopistoma wouterae Lieftinck, 1932: 44–55, pls 1–2. (orig.); Lafon, 1952: 433 (table); Peters, 1967: 211–213, figs 2, 8, 10, 16, 26 (redescription); Soldán & Braasch, 1984; 374–375 (key); Barber-James, 2009: 153–154, Table 2 (morph. matrix); Shi & Tong, 2013: 95 (key).

###### Material examined.

None.

###### Diagnosis.

The larvae of *P.wouterae* can be distinguished from those of other congeners by the combination of the following characteristics: (i) apical segment of the maxillary palp shorter than 1/2 the length of segment II, (ii) mesonotum with five dark-brown patches connected by brown narrow stripes, (iii) apex of the ventral margin of the fore-tibiae with eight serrated spines and (iv) posterolateral spine of segments VII and VIII parallel to or bent inwards the body axis (Shi and Tong 2013).

###### Distribution.

Songkhla province (S).

###### Remarks.

*Prosopistomawouterae* was originally described by Lieftinck (1932) from West Java. Parnrong et al. (2002) reported larvae of *P.wouterae* from the Songkhla province. In this study, we sampled in the same stream (Boripat Waterfall) but unfortunately, no specimens were found during our collection. This species seems to have a distribution limited only to the Sunda Islands and southern Thailand. Only four genes (16S, 18S, 28S rDNA, and H3) were sequenced for *P.wouterae* (Ogden and Whiting 2005), and no COI sequences were available for comparison.

##### Prosopistoma carinatum sp. n.

Taxon classificationAnimaliaEphemeropteraProsopistomatidae

http://zoobank.org/E4B47533-39E5-45D6-BFAA-BE6B5E681C7B

Figures 1D , 1F , 6 , 7 , 8 , 9 , 10 , 12E , 12F , 14

###### Material examined.

***Holotype***. THAILAND; Holotype, 1 mature larva, Kanchanaburi province, Thong Pha Phum, Ban Pra Chum Mai, 14°35'01.4"N, 98°34'54.3"E, 233 m, 11.IV.2015, B Boonsoong leg. (ZMKU); ***Paratypes***. 1 larva same data as holotype (MZL, GBIFCH00657926); 6 larvae same data as holotype (ZMKU); 1 mature larva, 8 larvae, Kanchanaburi province, Ban Pra Chum Mai, 14°35'01.4"N, 98°34'54.3"E, 233 m, 26.IV.2016, B Boonsoong leg. (ZMKU).

###### Additional material.

THAILAND; 1 larva, Nakhon Si Thammarat province, Lan Sa Ka, Khao Luang water fall, 8°28'08.30"N, 99°46'14.20"E, 533 m, 2.VII.2016, B Boonsoong leg. (ZMKU); 1 larva, Narathiwat province, Waeng district, Ai Sae, 5°47'45.9"N, 101°50'5.46"E, 64 m, 21.IV.2018, B Boonsoong leg. (ZMKU).

###### Distribution.

Kanchanaburi province (W), Nakhon Si Thammarat province (S), Narathiwat province (S).

###### Diagnosis.

The larvae of *Prosopistomacarinatum* sp. n. can be distinguished from those of other species by the combination of the following characteristics (Table 3): (i) carapace with two longitudinal ridges on each side of the midline on its surface (Fig. 1F), (ii) antenna 7-segmented, (iii) carapace with three pale-coloured depressions on each side, (iv) carapace with a very narrow flange width and strong convexity, (v) carapace with a typically brown colouration and a distally irregular (zig-zag) yellow pattern at 0.7 times the length of the carapace, (vi) carapace with circular scale-like structures, (vii) presence of three long serrated bristles at the base of the inner canine and (viii) ventral margin of the fore-tibia with nine pectinate setae.

**Table 3.** Larval characters of *P.carinatum* sp. n. compared with Thai known species (Soldán and Braasch 1984, Sartori and Gattolliat 2003, Shi and Tong 2013).

	* P. annamense *	* P. sinense *	* P. wouterae *	*P.carinatum* sp. n.
Distribution	Vietnam, Thailand	China, Thailand	Java, Sumatra, Thailand	Thailand
Antennae	5-segmented	5-segmented	6-segmented	7-segmented
Antenna with segment III to remaining segments	shorter	longer	shorter	shorter
Number of bristles on mandibles	3	4–6	3	3
Number of spines on foretibiae	4–6	6–8	8	9
Carapace flange width	wide	narrow	narrow	very narrow
Carapace ridges	no	no	no	2 ridges on each side
Distal end of carapace	incised notch	incised notch	incised notch	protruding notch
Length of inner canine to outer canine	shorter than outer canine	shorter than outer canine	subequal	subequal
Ratio length of carapace (along median suture):width	0.95–1.00 (n = 3)	0.96–1.02 (n = 3)	unknown	1.07–1.13 (n = 3)
Convexity: carapace depth:length	0.39–0.45 (n = 3)	0.30–0.49 (n = 3)	unknown	0.56–0.58 (n = 3)

###### Description

(in alcohol). Body length 1.94–2.43 mm excluding caudal filaments.

**Figure 6.**
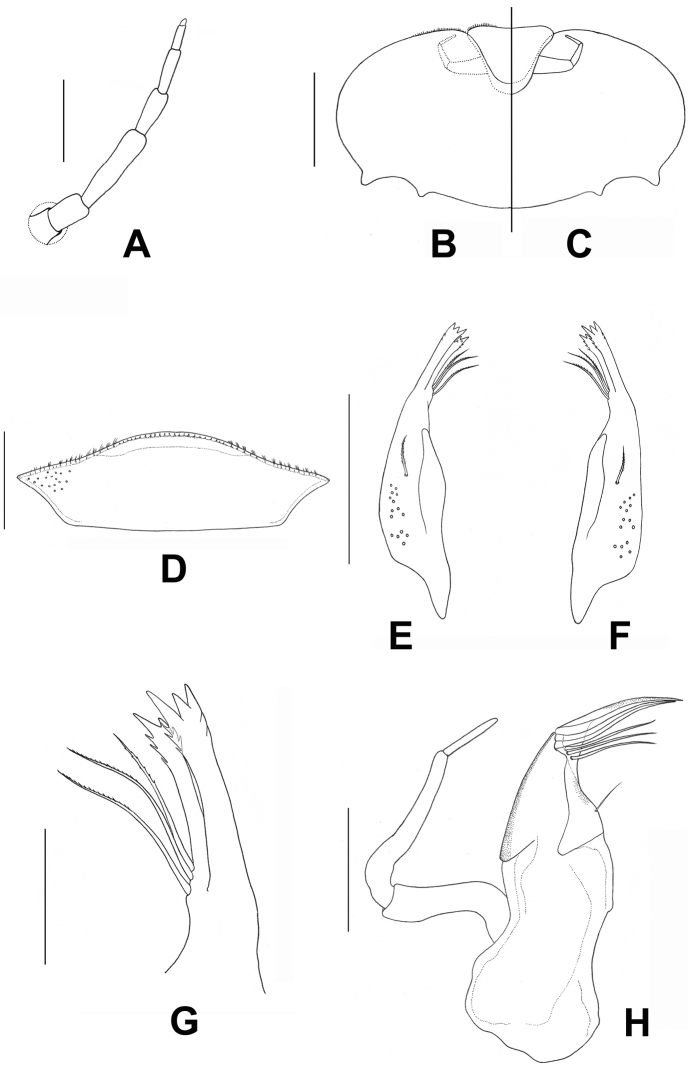
*Prosopistomacarinatum* sp. n.: **A** Antenna **B–C** Labium **B** ventral view **C** ventral view **D** Labrum, dorsal view **E–G** Mandible **E** Left mandible (ventral view) **F** Right mandible (ventral view) **G** Enlargement of canine of the right mandible, (**H**). Scale bars: 0.02 mm (**H**); 0.05 mm (**G**); 0.1 mm (**A, D, E, F**); 0.2 mm (**B, C**).

***Head.*** Head yellowish with median blackish ocellus between antennae (Fig. 8A), head width approximately 3 times length. Epicranial ecdysial sutures prominent, passing through the anterior margin of the lateral ocelli, and between the compound eyes and the antennal bases and continuing to the lateral margin of the head (Fig. 8B). Antenna 7-segments (Figs 6A, 8B, 8C), longer than the distance from antennal base to anterior margin of head; segment I usually retracts into head capsule; segment III the longest and shorter than combined length of segments IV–VII, antenna segment VII minute (Fig. 8C). Labrum (Fig. 6D) narrow, 3 times wider than long, anterior margin fringed with dense fine setae. Left and right mandibles similar (Fig. 6E, 6F). Outer canine of mandibles subequal and broader than inner canine, with three apical teeth, a smaller outer tooth and a larger inner tooth with a serrated margin near the apex and three small spines; inner canine with two apical teeth, including a larger inner one with a serrated inner margin near the apex with three spines. Three long serrated bristles arising from the base of the inner canine (Fig. 6G). A single stout feathered seta lateromedially on each mandible (Fig. 6E, 6F). Maxillae (Fig. 6H) crowned by a rigid canine and three subequal moveable dentisetae; three long-feathered stout bristles arising near the base of the apical canine and dentisetae on the galea-lacinia. A single unserrated bristle arising approximately 2/3 of the way down the sclerotised section of the galea-lacinia. Maxillary palpi 3-segmented, segment II the longest, length ratio of maxillary palp segments from basal one to apical: 1.9:2.2:1(Fig. 6H).

***Carapace.*** Carapace general colouration dark brown, distally with an irregular (zig-zag) yellow pattern at 0.7 times the length of the carapace (Fig. 1D). Two longitudinal ridges on the anterior surface region, and two short ridges on the pale surface area (Figs 1D, 1F, 9A, 9C, 9D). Carapace with one pale-coloured circular depression on lateral side of the anterior region (Figs 1F, 9B) and two pale-coloured striped depressions at the midline. Carapace flange width very narrow. Cuticle of carapace coarsely pitted and interspersed with scale-like structures (Fig. 9E, F). Distal margin of the carapace protruding slightly over the exhalent notch (Figs 1D, 9A). In lateral view, carapace strongly convex, with a convexity (ratio of maximum carapace height to length along the posterior margin of the carapace) range 0.56–0.58. Sternum pitted, with coarse scale-like structures within the triangular sternal plate (Fig. 8D, F).

**Figure 7.**
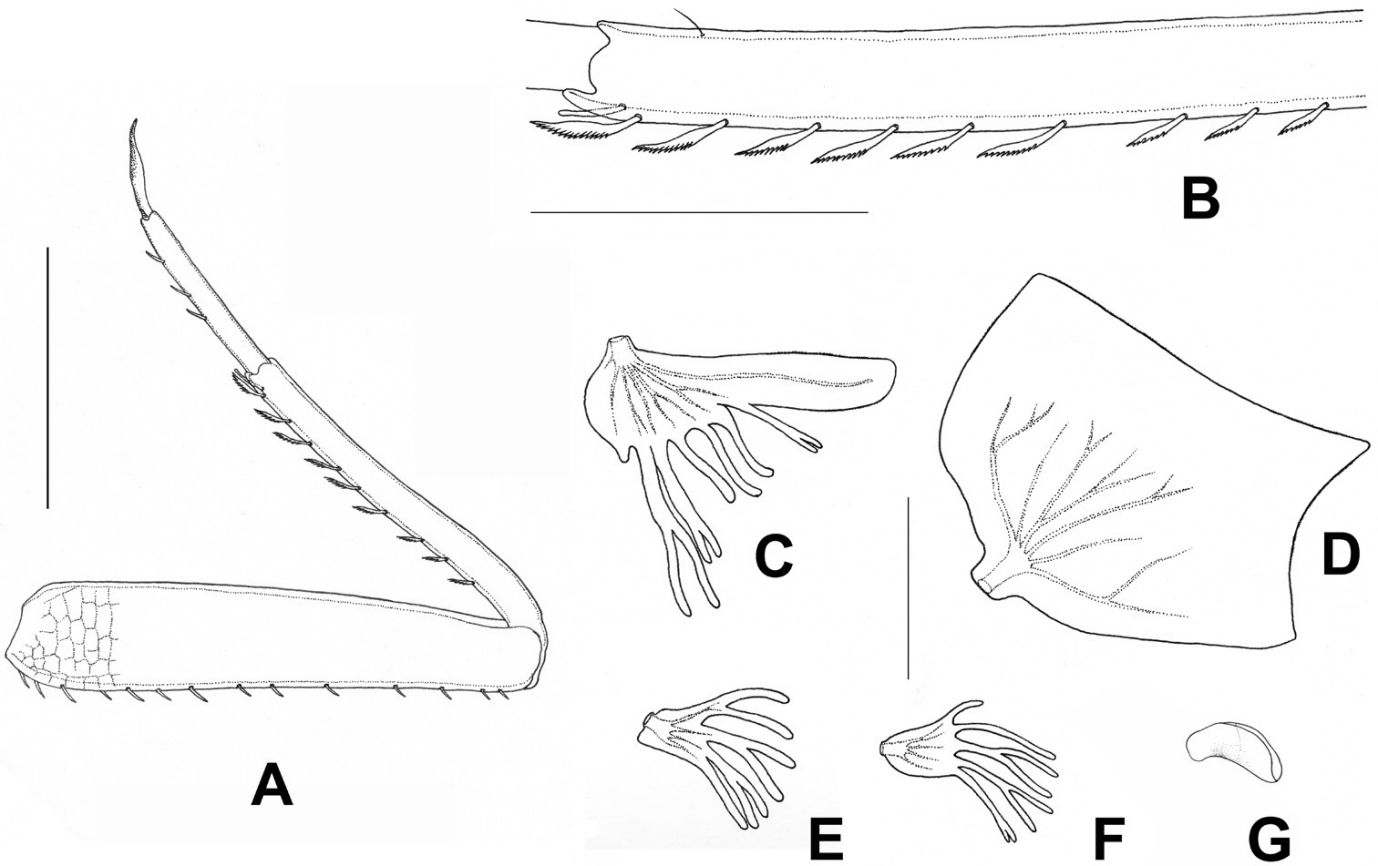
*Prosopistomacarinatum* sp. n.: (**A**) Foreleg, (**B**) Enlargement of part of the fore-tibia to show setation; (**C**) Gill I; (**D**) Gill II; (**E**) Gill IV; (**F**) Gill V; (**G**) Gill VI. Scale bars 0.1 mm (**B**); 0.2 mm (**A**); 1 mm (**C–F**).

***Legs.*** Dorsal margin of fore femur with 14 short simple setae (Fig. 7A); ventral margin of fore tibia with nine pectinate setae (Figs 7B, 10C). Anterior and posterior surface of femur covered with scale-like pattern (Figs 7A, 10A). Tarsal claws of all three pairs of legs sharp and without denticles.

***Abdomen.*** Abdominal gills (Fig. 7C–G). Gill I with lamellate upper portion, margin serrated, lower portion divided into multiple filaments (Fig. 7C). Gill II expanded to form broad leaf-like lamella (Fig. 7D). Gills III-V with multiple branching filaments (Fig. 7E, F). Gill VI tiny, unbranched (Fig. 7G). Posterolateral projections of abdominal segments VII–IX broad, apex pointed. Caudal filaments small, retractile, short, feathered.

**Figure 8.**
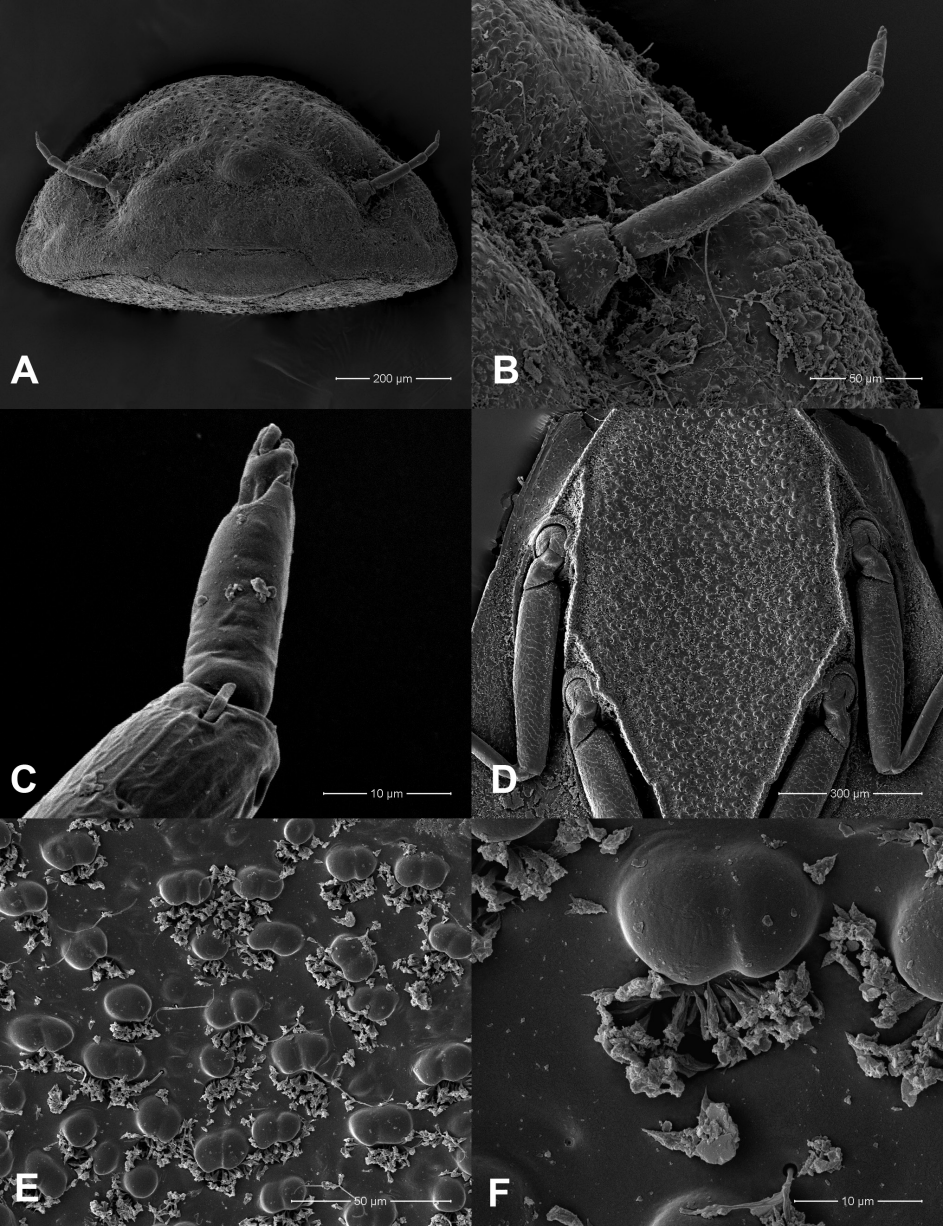
*Prosopistomacarinatum* sp. n.: **A**SEMs of head, frontal view **B** antenna, frontal view **C** tip of antenna **D** sternal plate and legs **E** closer view of surface of sternal plate **F** closer view showing details of scale-like structures.

**Figure 9.**
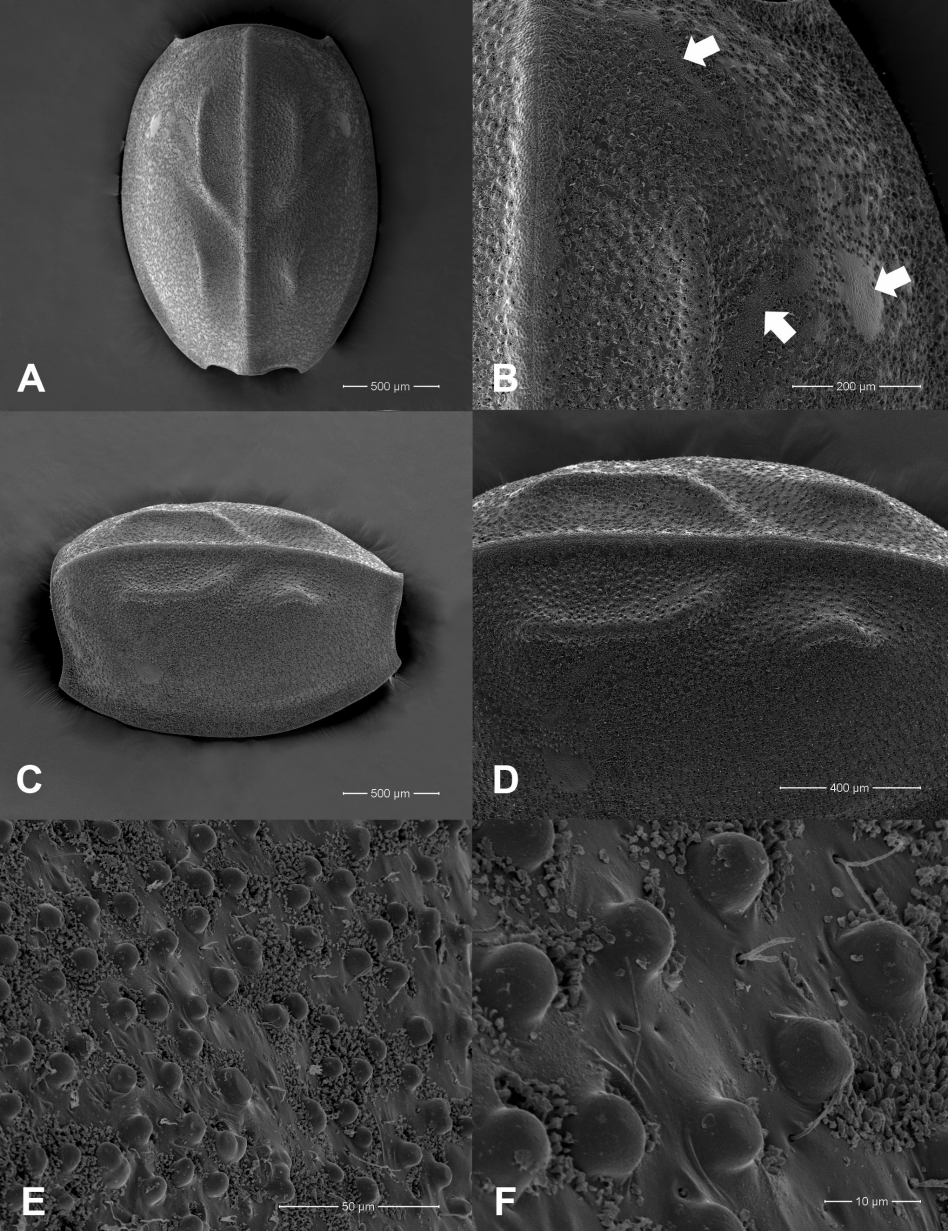
*Prosopistomacarinatum* sp. n.: **A**SEMs of notal shield, dorsal view **B** pale-coloured areas on carapace (arrows) **C** dorsolateral view of carapace **D** ridged longitudinal lines, dorsolateral view **E** scale-like structures **F** closer view showing details of scale-like structures.

**Figure 10.**
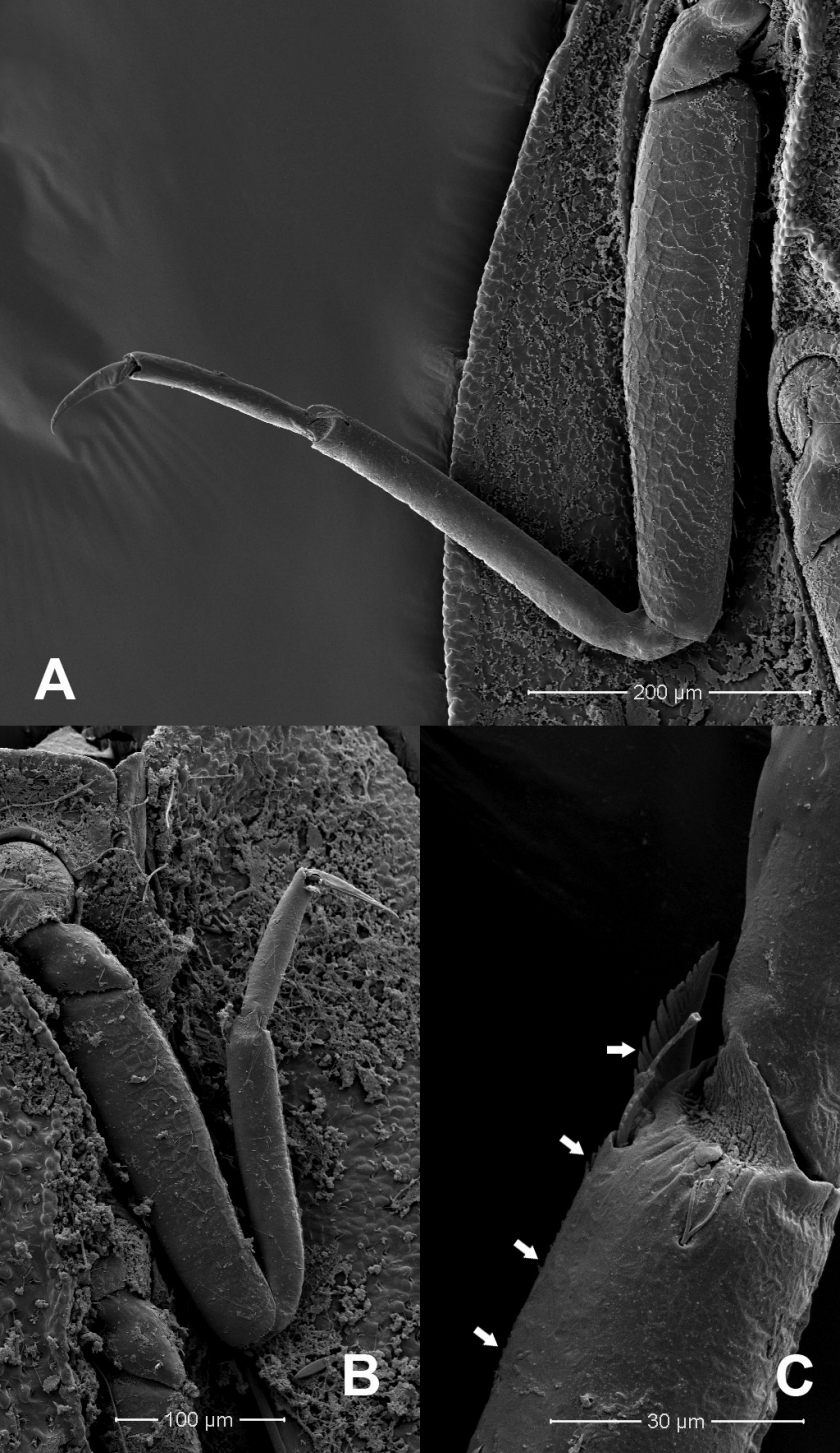
*Prosopistomacarinatum* sp. n.: **A**SEMs of middle leg showing scale-like pattern on femur, ventral view **B** ventral margin of fore tibia **C** pectinate setae (arrows).

###### Subimago and Imago.

Unknown.

###### Etymology.

The name *carinatum* (Latin for carinate or keeled), refers to the prominent keels or ridge-like mesonotal convexity of the species.

**Figure 11.**
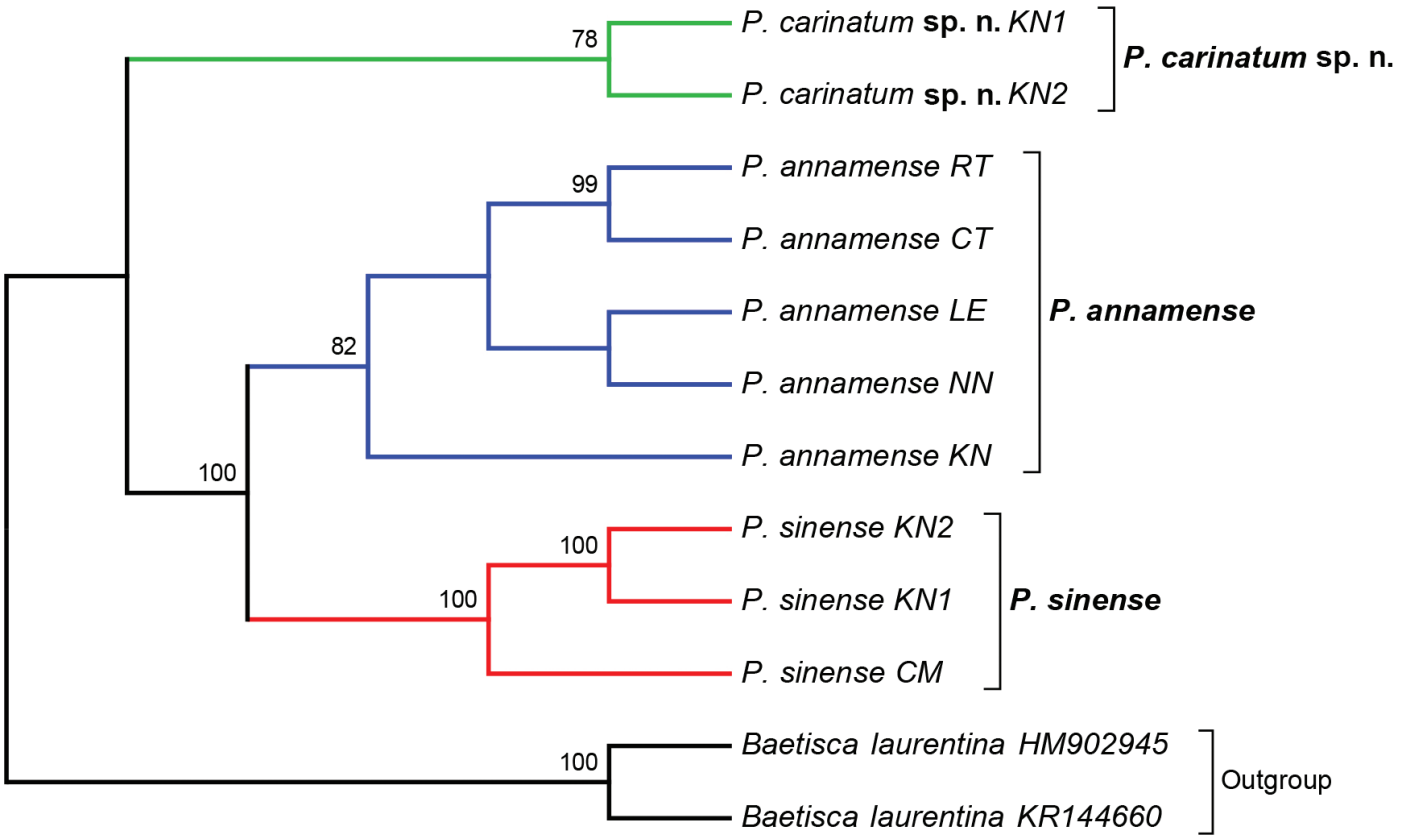
Consensus phylogenetic tree based on the maximum likelihood (ML) analysis of three Thai *Prosopistoma* species (the best model: T92 +G, parameter = 0.2619). The bootstrap consensus tree inferred from 1,000 replicates. Values above the branches are ML bootstrap values (> 70%). *Baetiscalaurentina* from GenBank was used as the outgroup. There were a total of 658 positions in the final dataset. Abbreviations are the same as those found in Table 1.

**Figure 13.**
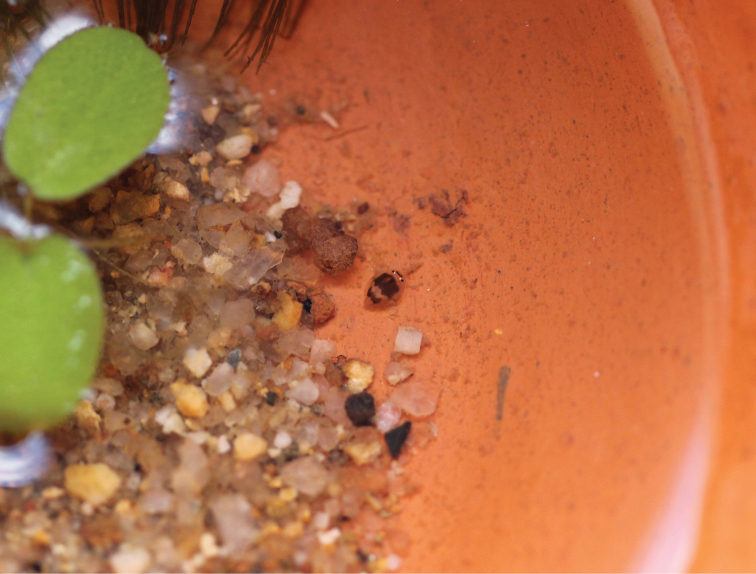
Rearing chamber of *Prosopistomaannamense* larva.

**Figure 12.**
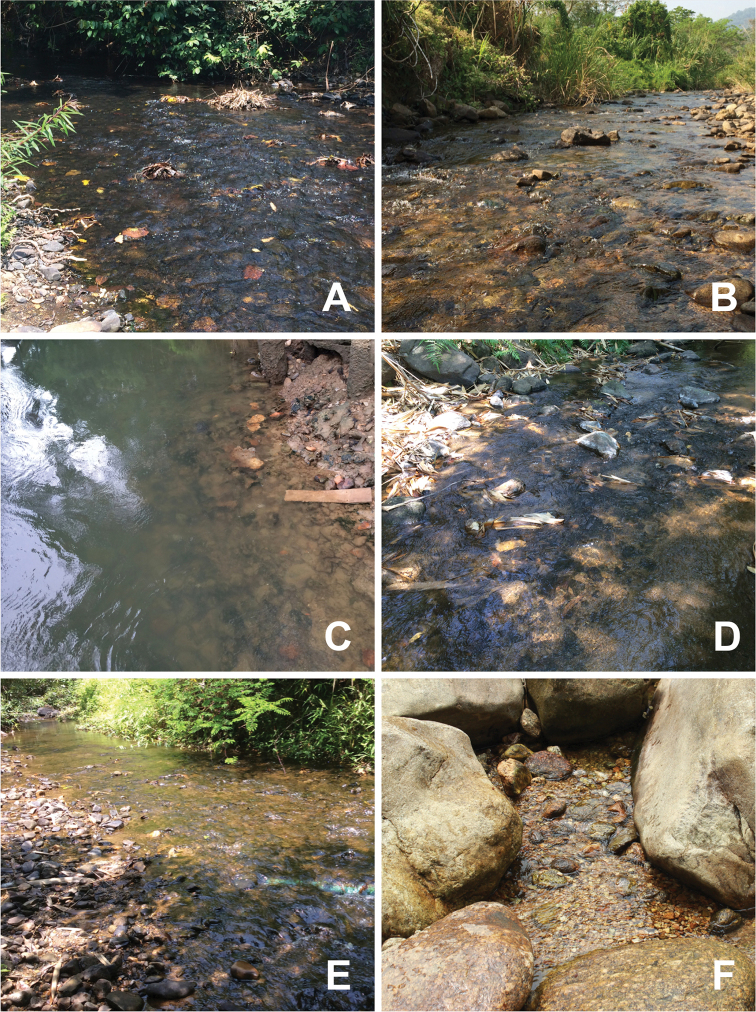
Habitats of *Prosopistoma* larvae: **A–C***P.annamense***A** = Loei province **B** = Nakhon Nayok province **C** = Kanchanaburi province (Khwae Noi River) **D***P.sinense* (Kanchanaburi province) **E–F***P.carinatum* sp. n. **E** = Kanchanaburi province **F** = Nakhon Si (Thammarat province).

**Figure 14.**
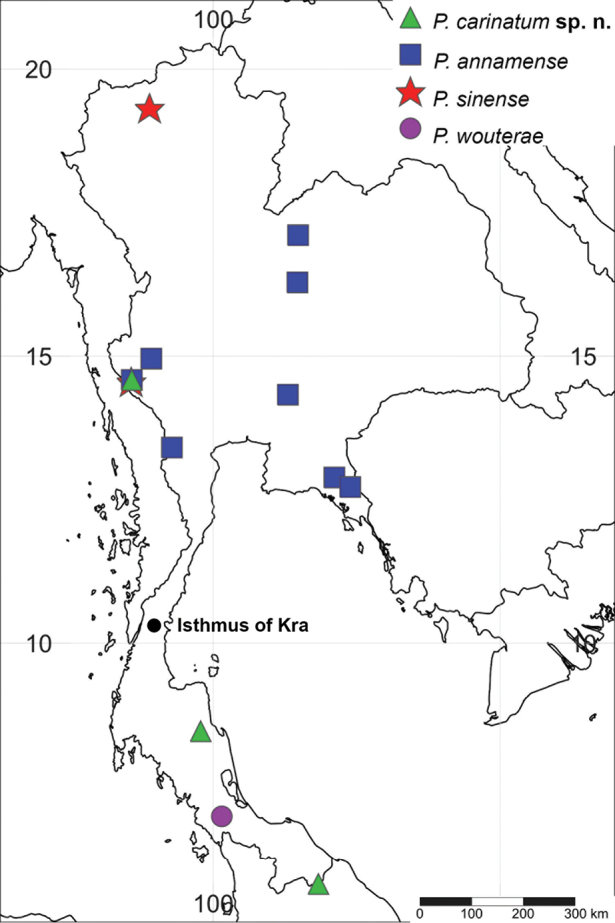
Distribution map of the four known Thai *Prosopistoma* species.

### Mitochondrial COI sequence analysis

The partial sequences of the mitochondrial COI gene (658 bp) of three species found in Thailand were analysed to investigate species delineation. Ten specimens of *Prosopistoma* were examined. In this study, we used *Baetiscalaurentina* from GenBank (HM902945 and KR144660) as the outgroup since Baetiscidae is the sister family of Prosopistomatidae (Ogden et al. 2009). The consensus phylogenetic tree of ML analysis is shown in Figure 11. The tree clearly showed a monophyletic clade for the new species *(P.carinatum* sp. n.). Clade 2 includes *P.annamense* and *P.sinense*. The interspecific genetic distance ranged from 31 to 35% among the investigated species (Table 2).

## Discussion

The molecular analysis revealed that *Prosopistomacarinatum* sp. n. is clearly separated from the other species. The molecular analysis suggests the morphological distinction between the three species. Morphologically, the new species differs from the two previously known species (*P.annamense* and *P.sinense*) by (i) longitudinal line ridges on the carapace, (ii) a strongly convex carapace, (iii) the sternal plate, (iv) the scale-like structures on the carapace, (v) 7-segmented antennae and (vi) nine pectinate setae on the ventral margin of the fore-tibiae. The larvae of *P.annamense* and *P.sinense* were placed in the same clade, and they share the characteristics of 5-segmented antenna and a smooth carapace. Moreover, the sternal plate of *P.annamense* and *P.sinense* has smooth surface (Figs 3A, 5A), while in *P.carinatum* sp. n. it bears distinctive scale-like structures (Fig. 8D–F). SEM examination on the carapace of the new species reveals a covering of circular scale-like structures scattered over the surface (Fig. 9E, 9F). The cuticle patterns of carapace of *P.annamense* is quite similar to *P.sinense* (Figs 2A–D, 4A–D), which are smoother than in the new species.

A comparison of the larvae of all Thai species is shown in Table 2. The irregular (zig-zag) pattern which occur on the carapace of *P.carinatum* sp. n. can also be found in *P.annamense* and *P.leftincki*. The comparison between the new species and other known Oriental species indicates close morphological similarity between *P.carinatum* sp. n. and *P.someshwarensis* (from India) in terms of the presence of a longitudinal line that looks like a ridge (Roopa et al. 2017). However, the new species can be easily distinguished by its two distinct longitudinal ridges.

## Ecology

The beetle-like mayfly larvae *Prosopistoma* inhabit shallow water under small stones in streams with a moderate to rapid current (Soldán and Braasch 1984, Sartori and Gattolliat 2003, Shi and Tong 2013). It is well documented that Prosopistomatidae are sensitive to pollution (Barber-James 2010). In this study, the habitats of the larvae were generally located in undisturbed upstream sites (forest streams), except *P.annamense*, which could be found in both disturbed and undisturbed sites. In addition, the larvae of *P.annamense* were also found in large rivers, including in the large urban river Xiangjiang in China (Yam 2015). In general, the larvae of *Prosopistoma* are rarely collected. In this study, most of the larvae were found from February through April in streams, with the exception of the southern region, in which they were found in July. *Prosopistomaannamense* larvae were found in October in large rivers. The ecological aspects of Thai *Prosopistoma*, such as microhabitat, feeding, life history and season of larval maturity, should be explored in more detail. In this study, the larvae *P.annamense* were reared in an earthenware pot with aquatic plants without an extra air supply (Fig. 13). Under these conditions, the larvae can grow to maturity and survive for approximately one month. However, the fully-grown larvae did not moult to the subimaginal stage.

In Thailand, the Isthmus of the Kra region is a widely recognised as a biogeographic boundary (Hughes et al. 2003, de Bruyn et al. 2005, Jantarit et al. 2013). The distribution of the Prosopistomatidae in Thailand is shown in Figure 14. *Prosopistomasinense*, which belongs to the ‘*P.variegatum*’ clade (Barber-James 2009), is distributed only in the north and west of Thailand, whereas *P.annamense* is widely spread through northeast, central, and west Thailand. However, neither *P.annamense* nor *P.sinense* were found on the other side of the isthmus. The distribution of *P.carinatum* sp. n. seems to overlap the isthmus region, and this species apparently falls into the ‘*African*’ clade. Barber-James (2009) examined the distribution of *P.wouterae* and suggested that there was a more recent dispersal from the Sunda Islands of this species after the sea level dropped. *Prosopistomawouterae* seems to be restricted to the south of the Isthmus of Kra.

## Supplementary Material

XML Treatment for Prosopistoma annamense

XML Treatment for Prosopistoma sinense

XML Treatment for Prosopistoma wouterae

XML Treatment for Prosopistoma carinatum
